# Children's and adolescents' snacking: interplay between the individual and the school class

**DOI:** 10.3389/fpsyg.2015.01308

**Published:** 2015-09-08

**Authors:** Helge Giese, Diana Tãut, Hanna Ollila, Adriana S. Baban, Pilvikki Absetz, Harald T. Schupp, Britta Renner

**Affiliations:** ^1^Department of Psychology, University of KonstanzKonstanz, Germany; ^2^Department of Psychology, Babeş-Bolyai UniversityCluj-Napoca, Romania; ^3^Department of Alcohol, Drugs and Addiction, National Institute for Health and WelfareHelsinki, Finland

**Keywords:** social norm, snack intake, food preference, self-concept, adolescents, eating, social environment

## Abstract

**Objective:** In schools, perceived norms of classmates facilitate but can also inhibit unhealthy food intake in children and adolescents. However, the role of actual class behaviors and attitudes is less established. Thus, the present study examined classmates' actual eating behavior and food preferences in relation to actual food intake. In addition, it tested whether these normative effects are facilitated by corresponding individual and class food preferences or a positive social self-concept.

**Methods:** The food preferences, social self-concept, and unhealthy snacking frequency of 734 Finnish, 829 German, and 555 Romanian children and adolescents (aged 8–19) from 127 school-classes were assessed.

**Results:** Multilevel analysis at individual and class level showed that classmates shared similar snacking habits (14.7% variance). Moreover, the unhealthy food preference of a school-class was associated with its collective snacking [$\chi_{\left(1\right)}^{2}=54.67$, *p* < 0.001, *PRV* = 0.32). This effect was facilitated by individual, unhealthy food preferences [$\chi_{\left(1\right)}^{2}=16.72$, *p* < 0.001, *PRV* = 0.57] and a positive social self-concept [$\chi_{\left(1\right)}^{2}=5.91$, *p* = 0.015, *PRV* = 0.12].

**Conclusions:** Actual class norms are related to children's and adolescents' eating, but their impact depends on individual differences in preferences and social self-concept.

## Introduction

In the exploration of eating behavior, social norms have been one focus of attention, particularly for adolescents (e.g., Stead et al., 2011; McEachan et al., 2011). Most research on the effects of norms on children's and adolescents' eating behavior has been conducted with regard to individual perception of norms, also called perceived descriptive norms (Woodward et al., 1996; De Bourdeaudhuij and van Oost, 2000; Cullen et al., 2001; Baker et al., 2003; Grimm et al., 2004; Vereecken et al., 2005; Thompson et al., 2007; Van der Horst et al., 2008; Lally et al., 2011; Giese et al., 2013). Exemplarily, Baker et al. (2003) observed that perceived peer norms were more strongly related to diet than parental norms. Furthermore, perceived norms of peers were assessed at the level of close friends, school peers, and out-of-school friends as well as across a range of food items, such as snacks, soft drinks, fruit, vegetables, and healthy and unhealthy foods. Reviewing the available literature, Stok et al. (under review) concluded that the majority of studies demonstrate a significant positive association between perceived norms and aspects of adolescent eating behavior.

These findings, however, do not clarify whether the perception of norms reflects what a norm group actually does or thinks (Rimal and Real, 2003; Stok et al. under review). Some studies show that perceptions of eating norms within school classes do not match the actual behavior based on behavioral reports by classmates (Perkins et al., 2010; Lally et al., 2011). This leads to the question, whether the portrayed norm-behavior relationship is mainly attributable to the perception or also to the actual behavior of relevant norm groups. To answer this question, the role of actual norms, i.e., the actual behavior and preferences by a group, needs to be examined for individual eating behavior. Corroborating the evidence for perceived norms, an association between actual norms based on behavioral reports and eating behavior has been found in studies in which close friends were the norm reference (De la Haye et al., 2010; Wouters et al., 2010; Ali et al., 2011). For instance, using data from a representative US sample of adolescents, Ali et al. (2011) observed a significant relationship between the actual behavior of best friends and the fast food consumption of individuals when controlling for individual, family, and school variables. In contrast to the positive findings observed for best friends, two studies examining actual classroom norms based on behavioral reports revealed no additional predictive value of actual norms when taking perceived norms into account (Perkins et al., 2010; Lally et al., 2011). Thus, studies on the relevance of actual norms are sparse, still inconclusive, and warrant more research and new approaches for determining their role in eating behavior of children and adolescents. To this end, this study examines both normative effects of the reported actual eating behavior of the school class (behavioral norms) and its preferences (preferential norms). As a selection, it focuses on normative effects of energy-dense, sweet and salty snack consumption. This special type of eating behavior appears especially suitable for studying eating norms in a classroom environment as it is quite frequent in schools (Grenard et al., 2013) and therefore meaningful for a school class (Terry et al., 2000; see also Wouters et al., 2010).

The type of food and the frequency of its consumption in schools might not be the only factors that possibly change the role of classroom norms. In addition, individuals might differ in how much their eating behavior is adjusted by actual peer norms. For instance, consistency between individual preferences and class norm might facilitate confirmatory behaviors (Terry et al., 2000). The rationale behind this so-called congruent consistency hypothesis (Acock and DeFleur, 1972) is that it should be easier to adhere to a norm, if an individual holds similar preferences. In contrast, the theory of reasoned action (Fishbein and Ajzen, 1975) predicts norm and preference to be independently predictive for intentions. Thus, this study also tests whether individual food preferences and actual class norms relate to food intake synergistically (i.e., consistent unhealthy preferences increase intake above additive effects) (Acock and DeFleur, 1972; Povey et al., 2000; Terry et al., 2000) or additively (i.e., both increase intake independently) (Fishbein and Ajzen, 1975).

Another individual difference that changes norm relevance might be the perception of acceptance by others, also called social self-concept (Berndt and Burgy, 1996). This self-concept is especially interesting if group norms and individual preferences are in conflict: On the one hand, high perceived acceptance might indicate higher norm compliance. On the other hand, a positive social self-concept might also denote high individual self-esteem and allows pursuing rather personal goals (e.g., Leary et al., 1995; Williams, 2009). Therefore, it is promising to regard the interplay of social self-concept with both group and individual preferences when evaluating children's and adolescents' eating behavior.

In conclusion, the present study examined the role of actual classroom norms in children's and adolescents' snack consumption. Specifically, the study investigated the role of actual behavioral and preferential norms based on behavioral reports. Furthermore, potential individual differences in the effects of actual norms were tested. To this end, the role of individual preferences, social self-concept, and the interplay of both effects were considered. To ensure a broad cultural context, these analyses were conducted with a large and diverse sample of school classes from Germany, Finland, and Romania.

## Methods

### Participants and procedure

In the winter of 2010, 734 Finnish, 829 German, and 555 Romanian children and adolescents aged 8–19 (55.9% female) completed a survey in their classroom (for a detailed description see Giese et al., 2015). All participants, their parents, and governmental institutions gave informed consent prior to answering. The study was conducted in 127 classes^1^ with a mean of 16.76 students per class (Range: 1–64 students; *Mdn* = 16; *SD* = 10.76). Classes were from both rural and urban backgrounds. The survey included demographic variables, food preferences, social self-concept, and habitual snack intake. The questionnaire was developed in English as a “mother version” and translated into the respective languages by native-speakers. Comparability of meaning across countries was ensured by reverse translations into English. This study was part of the EU-project TEMPEST [Health-F2-2008-223488], which investigated eating behavior in children and adolescents.

### Measures

For multilevel data, it is important to note that the meaning of a variable depends on the data aggregation level (individual vs. class level) (e.g., Robinson, 1950). For example, preferences at the individual level reflect how much a participant prefers unhealthy foods. In contrast, preference on the class level is based on the group mean of each class and thus reflects how much the class as a whole prefers unhealthy food. Therefore, we chose different terms for each measure at each level of aggregation. As such, food preference is termed “individual preference” at the individual level and “class preference” as an aggregate at the class level. In order to achieve this separation of individual from class level predictors, all variables were group-mean centered for individual-level predictors, and grand-mean centered group-means were computed as class level variables (c.f. Enders and Tofighi, 2007). Binary class level variables (gender ratio, Finnish and German class identity) were effect coded. Due to group mean centering, all individual scores are expressed relative to the classmates' scores as fluctuation around the class mean. In addition, group mean centering of individual level variables enables the interpreting of all class level effects independently from individual-level effects for all variables (e.g., Enders and Tofighi, 2007).

#### Social affluence

Social affluence was assessed using a measure adapted from Wößmann (2003). Based on the Third International Mathematics and Science Study (TIMSS), Wößmann (2003) has suggested the number of books in the participants' home as a proxy for the educational, social, and economic background of the participants' families that is more readily comparable across countries. Participants were asked to report the approximate number of books in their household (excluding school books, magazines, and newspapers). Response categories included (1) “no/few books (0–10),” (2) “one bookshelf (11–25),” (3) “one bookcase (26–100),” (4) “two bookcases (101–200),” and (5) “more than two bookcases (more than 200).” The individual level of this measure is termed “individual affluence” and the class level “class affluence.”

#### Food preference

Food preference for food items high in energy density or low nutritional value (“unhealthy”) vs. food items low in energy density or high in nutritional value (“healthy”) was assessed in a dichotomous choice task including 22 food pairs (c.f. Calfas et al., 1991; Giese et al., 2013). Participants could choose between an unhealthy and a healthy food item illustrated using photographs for 10 pairs of snacks (e.g., chocolate vs. apple), four pairs of meals (e.g., pizza vs. vegetable soup) and eight pairs of beverages (e.g., sugared soft drink vs. water) with the instruction, “If you could choose a …, what would you choose?” The food items were not labeled as “unhealthy” or “healthy” to avoid effects of negative framing (see also Lally et al., 2011). For the food choice task, two food items of the same food type category were presented. Thus, participants could only choose between snacks, or meals, or beverages. Choices across food categories, e.g., choosing pizza over an apple, were not possible. The total of all unhealthy choices across the 22 food pairs indicates preferences for unhealthy food items compared to healthy ones. The two levels of preferences were separated into “individual” and “class preferences.”

#### Social self-concept

In a first step, focus groups were conducted in separate school classes to identify which concepts the children and adolescents associated with popularity and group-acceptance. Based on these results and a literature review (e.g., LaFontana and Cillessen, 2002), seven social concepts were selected reflecting participant's perception of their acceptance. Each participant rated him- or herself on these seven aspects on a seven-point semantic differential scale (“uncool” vs. “cool,” “unpopular” vs. “popular,” “unattractive” vs. “attractive,” “I never get to decide” vs. “I get to decide,” “Others never listen to me” vs. “Others listen to me,” “I have few friends” vs. “I have a lot of friends,” and “I am not liked” vs. “I am liked”). As a measure of social self-concept, the seven items were averaged with (1) indicating a negative and (7) a positive social self-concept. Here, the individual level is labeled “social self-concept,” whereas the class level is labeled “class cohesion” as it describes how much the class as a whole feels accepted.

#### Unhealthy snack intake

Intake of snacks high in energy density or of low nutritional value, that is sweet and salty snacks, was assessed by a food frequency questionnaire (FFQ) (e.g., Willett et al., 1985), which has been shown to be applicable and valid for children and adolescents (Rockett et al., 1997). Participants indicated how often they ate (1) candy, (2) savory snacks, (3) chocolate bars/chocolate, (4) cookies, (5) cake, and (6) ice cream on a 7-point scale [(0) “never,” (0.25) “less than once a week,” (1) “once a week,” (3) “2–4 days a week,” (5.5) “5–6 days a week,” (7) “every day, once a day,” (8) “every day, more than once”]. All items were recoded and averaged to yield the weekly consumption frequency of each snack. “Unhealthy snacking” is referred to as snacking on the individual level and “collective snacking” on the class level.

### Statistical analysis

In a first analysis, the relevance of behavioral class norms for reported unhealthy snacking was assessed. This was achieved by testing behavioral similarity of class members via an intercept only model. The intercept only model separates snacking into individual snacking and collective snacking variance. Thus, it can be applied to estimate the proportion of variance explained by class membership compared to the total amount of variance. This proportion is referred to as intraclass correlation (ICC). High unhealthy snacking similarity among classmates can be interpreted as a sign of a behavioral norm. In order to exclude alternative explanations, the ICC was also estimated, controlling for socio-demographical variables at all levels (**Table 2**, Model 2 and 3). In addition, further clustering was considered for members of each school and same-sex classmates (**Table 2**, Model 3a and 3b). Moreover, equality of ICCs across girls and boys in each class, children (class average younger than 14) and adolescents, and countries was tested in Mplus7.

In a second analysis, after testing the behavioral norm effects via ICCs, the effects of class preferences as preferential norms were tested. The comparison to the behavioral norm serves as validation for both measures. Applying preferential norms furthermore enables to test for individual differences in class norms effects. This was achieved by modeling the frequency of unhealthy snacking in a multistep multilevel approach recommended by Hox (2010) (see **Table 3** for detailed order of steps): (1) starting again with an intercept only model, (2) fixed individual-level effects were tested followed by (3) class level effects, (4) random individual-level effects, and (5) cross-level interactions. For fixed effects, as in traditional regression approaches, control variables were introduced before variables of interest. Each step was tested by a χ^2^-test and AIC and in the final model, effects were evaluated by z-scores (see **Table 3**). The steps of analysis, which are important for our hypotheses, are highlighted below.

First, to validate class preference as preferential norm, its association with collective snacking as behavioral norm must be evaluated (step 3b). This preferential norm was then utilized in further analyses to investigate individual differences in actual normative class effects.

To this end, cross-level interactions are of central interest. Thus, the Individual preference × Class preference interaction was used to evaluate synergistic individual and group preference effects, which go beyond the additive main effects of both constructs (step 5a). To rule out other kinds of synergistic individual and class preference effects, quadratic effects of the variables individual preference and class preference and their interactions were included (this test is not included in **Table 3**). After this check, a Social self-concept × Class preference interaction was applied to test whether norms might be moderated by the social self-concept (step 5b).

Furthermore, testing for Individual preference x Social self-concept effects examined the independence of individual preference and social self-concept effects (step 2d). For further illustration, the Individual preference × Social self-concept × Class preference interaction was added to the final model (step 5c) and simple effects analyses were conducted according to Preacher et al. (2006).

If not mentioned otherwise, effects were tested with all complete cases in a multilevel regression approach (HLM) using SPSS 21 maximum likelihood estimates. Descriptive statistics were estimated by applying MLR estimators in Mplus7 (see Table 1). For reliability, omegas were assessed according to Bolger and Laurenceau (2013).

**Table 1:** **Descriptive Statistics**.

	***M***	***SD*_individual_**	***SD*_class_**	**ICC**	**ω_individual_**	**ω_class_**
Age	13.06	0.49	2.14	0.95		
Sex				0.08		
Social affluence	3.27	1.07	0.49	0.17		
Food preference	11.39	4.39	1.91	0.16	0.71	0.94
Social self-concept	5.57	0.89	0.32	0.11	0.91	0.99
Snack intake	1.77	1.51	0.61	0.14	0.87	0.94

## Results

### Actual behavioral and preferential norms

The intercept only model indicated similarity between classmates' unhealthy snacking. Specifically, 14.7% of the total variance of unhealthy snacking frequency was explained by class membership [$\chi_{\left(1,N\;=\;2118\right)}^{2}=128.34$, *p* < 0.001; z_*Wald*_ = 5.09; Table 2, Model 1]. Controlling for socio-demographical differences, class variance remained significant with a proportion of about 7.3% [$\chi_{\left(1,N\;=\;2118\right)}^{2}=41.33$, *p* < 0.001; z_*Wald*_ = 3.76; Model 2]. Moreover, no significant similarity remained at either school [$\chi_{\left(1,N\;=\;2118\right)}^{2}=0.13$, *p* = 0.716; z_*Wald*_ = 0.35; Model 3a] or same-sex classmate level [$\chi_{\left(1,N\;=\;2118\right)}^{2}=3.12$, *p* = 0.077; z_*Wald*_ = 1.46; Model 3b]; both were therefore excluded from the remaining analyses. Further tests determined that the ICCs were similar in all three countries [$\chi_{\left(2,N\;=\;2118\right)}^{2}=0.52$, *p* = 0.773], when comparing children and adolescents [$\chi_{\left(1,N\;=\;2118\right)}^{2}=2.641$, *p* = 0.104], and girls and boys [$\chi_{\left(1,\;N\;=\;2118\right)}^{2}=0.11$, *p* = 0.741].

**Table 2:** **Multilevel regression model of snacking illustrating behavioral norms**.

**Parameter**	**Model 1**		**Model 2**		**Model 3**		**Model 3a**		**Model 3b**	
**FIXED EFFECTS**										
Intercept	1.77^***^	(0.07)	1.77^***^	(0.05)	1.77^***^	(0.05)	1.78	(0.06)	1.77^***^	(0.05)
**Level 1 (Individual)**										
Age					−0.01	(0.07)	−0.01	(0.07)	−0.01	(0.07)
Gender					0.13	(0.07)	0.13	(0.07)	0.13	(0.08)
Individual affluence					−0.07^*^	(0.03)	−0.07^*^	(0.03)	−0.07^*^	(0.03)
**Level 2 (Class)**										
Age level			0.07^*^	(0.03)	0.07^*^	(0.03)	0.06^*^	(0.03)	0.07^*^	(0.03)
Gender ratio			0.29	(0.27)	0.29	(0.27)	0.27	(0.28)	0.29	(0.28)
Class affluence			−0.30^**^	(0.09)	−0.30^**^	(0.09)	−0.30^**^	(0.10)	−0.30^**^	(0.10)
Finnish class			−0.44^***^	(0.07)	−0.45^***^	(0.07)	−0.45^***^	(0.08)	−0.44^***^	(0.07)
German class			0.24^**^	(0.07)	0.24^**^	(0.07)	0.25^**^	(0.08)	0.24^**^	(0.08)
**RANDOM EFFECTS**										
Residual _within_	2.27^***^	(0.07)	2.28^***^	(0.07)	2.27^***^	(0.07)	2.27^***^	(0.07)	2.23^***^	(0.07)
Intercept _class_	0.39^***^	(0.08)	0.18^***^	(0.05)	0.18^***^	(0.05)	0.17^**^	(0.05)	0.15^**^	(0.05)
Intercept _school_							0.01	(0.04)		
Intercept _samegender_									0.07	(0.05)
$\chi_{\text{model}}^{2}$	128.34^***^		50.13^***^		8.99^*^		0.13		3.12	
*df*	1		5		3		1		1	
$\chi_{\text{interceptclass}}^{2}$ (*df* = 1)	128.34^***^		41.33^***^		42.16^***^		26.51^***^		8.33^**^	
AIC	7909.76		7869.63		7866.64		7868.51		7865.52	
ICC _class_ (in %)	14.70		7.29		7.39		7.02		6.12	

*Standard Errors are in parenthesis. The $\chi_{\text{model}}^{\text{2}}$-test refers to the change between the described model and the previous model (Model 3 for 3a and 3b). $\chi_{\text{interceptclass}}^{\text{2}}$ refers to the comparison of models with and without random class intercepts. The ICC is only computed for class level variance. p-values are two-tailed except for variance components*.

*** p < 0.001;

** p < 0.01

* *p < 0.05*.

After showing signs of similarity in unhealthy snacking between classmates as a form of behavioral norm, further analyses revealed that snacking was associated with preferential class norms as reflected by shared class preferences: for each collectively preferred additional unhealthy item, class intake frequency of each unhealthy snack increased by 0.2 per week (95% CI [0.16–0.25]). As such, after taking into account demographic variables (step 3b, Table 3), class preference explained 31.6% of collective snacking.

Table 3**Multilevel regression model of snacking illustrating preferential norms**.

**Table:** 

**Parameter**	**Step1**		**Step 2a**		**Step 2b**		**Step 2c**		**Step 2d**	
**FIXED EFFECTS**										
Intercept	1.77^***^	(0.07)	1.77^***^	(0.07)	1.77^***^	(0.07)	1.77^***^	(0.07)	1.77^***^	(0.07)
**Level 1 (Individual)**										
Age			−0.00	(0.07)	0.05	(0.06)	0.02	(0.06)	0.03	(0.06)
Gender			0.13	(0.07)	−0.14^*^	(0.07)	−0.15^*^	(0.07)	−0.15^*^	(0.07)
Individual affluence			−0.07^*^	(0.03)	−0.02	(0.03)	−0.02	(0.03)	−0.01	(0.03)
Individual preference					0.13^***^	(0.01)	0.13^***^	(0.01)	0.13^***^	(0.01)
Social self-concept							0.20^***^	(0.03)	0.19^***^	(0.03)
Individual preference × Social self-concept									0.03^***^	(0.01)
**Level 2 (Class)**										
Age level										
Gender ratio										
Class affluence										
Finnish class										
German class										
Class preference										
Class cohesion										
Class preference × Individual preference										
Class preference × Social self-concept										
Class preference × Social self-concept × Individual preference										
**RANDOM EFFECTS**										
Residual _within_	2.27^***^	(0.07)	2.26^***^	(0.07)	1.94^***^	(0.06)	1.91^***^	(0.06)	1.90^***^	(0.06)
Intercept _class_	0.39^***^	(0.08)	0.39^***^	(0.08)	0.43^***^	(0.08)	0.43^***^	(0.08)	0.43^***^	(0.08)
Individual preference										
Social self-concept										
Intercept with individual preference										
Intercept with social self-concept										
Individual preference with social self-concept										
χ^2^	128.34^***^		9.08^*^		301.22^***^		30.91^***^		12.92^***^	
*df*	1		3		1		1		1	
AIC	7909.76		7906.68		7607.46		7578.56		7567.64	
*PRV*^a^			0.005		0.141		0.013		0.005

**Table:** 

**Parameter**	**Step3a**		**Step 3b**		**Step3c**		**Step 4**		**Step5a**		**Step 5b**		**Step5c**	
**FIXED EFFECTS**														
Intercept	1.78^***^	(0.06)	1.77^***^	(0.04)	1.75^***^	(0.04)	1.76^***^	(0.04)	1.76^***^	(0.04)	1.76^***^	(0.04)	1.76^***^	(0.04)
**Level 1 (Individual)**														
Age	0.03	(0.06)	0.03	(0.06)	0.03	(0.06)	0.02	(0.06)	0.02	(0.06)	0.02	(0.06)	0.02	(0.06)
Gender	−0.15^*^	(0.07)	−0.15^*^	(0.07)	−0.15^*^	(0.07)	−0.16^*^	(0.07)	−0.17^*^	(0.07)	−0.16^*^	(0.07)	−0.17^*^	(0.07)
Individual affluence	−0.01	(0.03)	−0.01	(0.03)	−0.01	(0.03)	−0.00	(0.03)	−0.00	(0.03)	−0.00	(0.03)	−0.00	(0.03)
Individual preference	0.13^***^	(0.01)	0.13^***^	(0.01)	0.13^***^	(0.01)	0.13^***^	(0.01)	0.13^***^	(0.01)	0.13^***^	(0.01)	0.13^***^	(0.01)
Social self-concept	0.19^***^	(0.04)	0.19^***^	(0.04)	0.19^***^	(0.04)	0.21^***^	(0.04)	0.20^***^	(0.04)	0.20^***^	(0.04)	0.20^***^	(0.04)
Individual preference × Social self-concept	0.03^**^	(0.01)	0.03^**^	(0.01)	0.03^**^	(0.01)	0.03^**^	(0.01)	0.03^**^	(0.01)	0.03^**^	(0.01)	0.03^**^	(0.01)
**Level 2 (Class)**														
Age level	0.06^*^	(0.03)	−0.02	(0.02)	−0.03	(0.02)	−0.03	(0.02)	−0.03	(0.02)	−0.03	(0.02)	−0.03	(0.02)
Gender Ratio	0.24	(0.28)	0.13	(0.22)	0.00	(0.22)	0.11	(0.21)	0.08	(0.21)	0.07	(0.21)	0.10	(0.21)
Class affluence	−0.29^**^	(0.10)	−0.20^*^	(0.08)	−0.19^*^	(0.07)	−0.13	(0.07)	−0.13	(0.07)	−0.13	(0.07)	−0.13	(0.07)
Finnish class	−0.44^***^	(0.08)	−0.23^***^	(0.06)	−0.11	(0.07)	−0.13	(0.07)	−0.13	(0.07)	−0.12	(0.07)	−0.13	(0.07)
German class	0.24^**^	(0.08)	−0.03	(0.07)	−0.01	(0.07)	−0.03	(0.06)	−0.03	(0.06)	−0.02	(0.06)	−0.02	(0.06)
Class preference			0.19^***^	(0.06)	0.20^***^	(0.02)	0.18^***^	(0.02)	0.19^***^	(0.02)	0.20^***^	(0.02)	0.20^***^	(0.02)
Class cohesion					0.47^**^	(0.14)	0.31^*^	(0.13)	0.34^*^	(0.13)	0.35^*^	(0.13)	0.34^*^	(0.13)
Class preference × Individual preference									0.02^***^	(0.00)	0.02^***^	(0.00)	0.02^***^	(0.00)
Class preference × Social self-concept											0.05^*^	(0.02)	0.05^*^	(0.02)
Class preference × Social self-concept × Individual preference													0.01	(0.00)
**RANDOM EFFECTS**														
Residual _within_	1.90^***^	(0.06)	1.91^***^	(0.06)	1.91^***^	(0.06)	1.80^***^	(0.06)	1.81^***^	(0.06)	1.80^***^	(0.06)	1.80^***^	(0.06)
Intercept _class_	0.22^***^	(0.05)	0.08^**^	(0.03)	0.07^**^	(0.03)	0.09^**^	(0.03)	0.09^**^	(0.03)	0.09^**^	(0.03)	0.09^**^	(0.03)
Individual preference							0.00^*^	(0.00)	0.00	(0.00)	0.00	(0.00)	0.00	(0.00)
Social self-concept							0.07^**^	(0.03)	0.07^**^	(0.03)	0.06^*^	(0.03)	0.07^*^	(0.03)
Intercept with individual preference							0.01	(0.01)	0.01	(0.00)	0.01	(0.00)	0.01	(0.00)
Intercept with social self-concept							0.06^*^	(0.02)	0.05^*^	(0.02)	0.05^*^	(0.02)	0.04^*^	(0.02)
Individual preference with social self-concept							−0.00	(0.00)	−0.00	(0.00)	−0.00	(0.00)	−0.00	(0.00)
χ^2^	46.95^***^		54.67^***^		11.39^***^		32.37^***^		16.72^***^		5.91^*^		1.94	
*df*	5		1		1		5		1		1		1	
AIC	7530.68		7478.01		7468.62		7446.25		7431.52		7427.61		7427.67	
*PRV*^a^	0.488		0.316		0.021				0.568		0.117			

*Standard Errors are in parenthesis. The χ^2^-test refers to the change between the described model and the previous model*.

a *PRV is the additional proportion of explained variance considering within variance for individual, between variance for class-level, and slope variance for cross-level interaction effects, as defined in Raudenbush and Bryk (2002) (c.f. Hox, 2010), and must be interpreted with caution. p-values are two-tailed except for variance components*.

*** p < 0.001;

** p < 0.01;

* *p < 0.05*.

### Individual differences in actual group norm effects: The role of individual food preferences and social self-concept

In order to test whether consistent individual and class preferences were associated with unhealthy snacking additively or synergistically, the interaction of individual preference and class preference was evaluated. Results indicated that individual preference moderated class preference effects on participant's unhealthy snacking behavior, explaining 56.8% of individual preference slope variance between classes (step 5a, Table 3). Specifically, the unhealthier the individual preference, the more snacking was associated with class preferences (95% CI [0.01–0.02]). Quadratic terms of class preference and individual preference and their interactions yielded no additional effects [$\chi_{\left(5,N\;=\;2118\right)}^{2}=6.11$, *p* = 0.296, AIC = 7435.41, all *b* ≤ |0.01|, all *p* ≥ 0.091]. Therefore, unhealthier individual preferences increased the relevance of class preferences regardless of whether they were healthy or unhealthy.

The interaction of class preference and social self-concept examined the hypothesis that positive and negative self-concept moderate the relevance of class norms. Results indicated that the effects of class preferences on individual unhealthy snack intake were moderated by participants' social self-concept, accounting for 11.7% of social self-concept slope variance between classes (step 5b, Table 3; 95% CI [0.01–0.09]). The more positive the social self-concept of children and adolescents compared to their classmates, the more they were coherent with the class preference.

Further analyses explored whether social self-concept and individual preference effects were dependent on each other. Results showed that the increasing effect of unhealthy individual preferences on unhealthy snacking was amplified in participants with a positive social self-concept (step 2d, Table 3; 95% CI [0.01–0.04]). Further tests revealed that the three-way interaction between individual preferences, social self-concept, and class preferences was not significant (step 5c, Table 3; 95% CI [-0.002 to 0.013]). Thus, social self-concept moderated the effects of individual and class preference, but not the interaction between the two variables.

**Figure 1:**
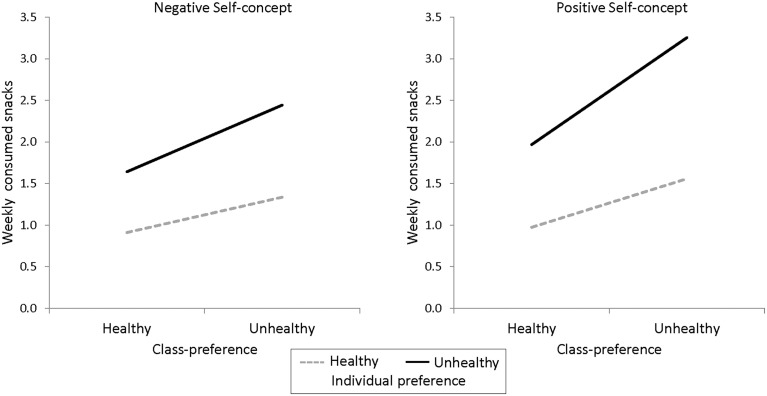
**Simple effects of class preference**. Simple slope effects for |1SD| in each variable. The snacking frequency refers to snacking of each single snack.

To illustrate the different interaction effects, a simple slope analysis (see Figure 1) was conducted. This analysis yielded that unhealthy class preferences were associated with increased unhealthy snacking most in individuals with unhealthy food preferences and a positive social self-concept (*b* = 0.34, *z* = 8.69, *p* < 0.001). Conversely, class preferences were less closely related to snacking for participants with unhealthy preferences and a negative social self-concept, (*b* = 0.21, *z* = 5.89, *p* < 0.001). Class preference effects on unhealthy snacking further decreased if an individual preferred healthy food regardless of social self-concept (positive: *b* = 0.15, *z* = 4.13, *p* < 0.001; negative: *b* = 0.11, *z* = 3.23, *p* = 0.001). That is, individuals consumed more unhealthy snacks if both the individual and the class preferred unhealthy foods, and this increased under the condition of a positive social self-concept. Yet, if the class and the individual preferred healthy foods, individuals consumed the least unhealthy snacks, irrespective of social self-concept (Figure 1).

## Discussion

Previous research indicates that the eating behavior of children and adolescents is associated with the actual behavior of close peers and friends based on behavioral reports (e.g., Ali et al., 2011). Extending this line of research, the present study examined the relationship of reported actual norms and eating behavior at the level of the school class. The data showed that the consumption frequency of unhealthy snacks is similar among school peers. Furthermore, class preferences for unhealthy foods as preferential norm were associated with similarity in unhealthy snacking. Contributing to existing research, inter-individual differences in the association of individual eating behavior and peers' actual norms could be shown. Specifically, class preference for unhealthy food items was related to an increased unhealthy snacking, particularly when the children and adolescents preferred unhealthy food items and had a positive self-concept.

A main finding of the present study is that class behavior and preference for healthy and unhealthy food items is reflected in the individual snacking behavior of children and adolescents. These results appeared robust when taking social affluence, age, and gender into account and were similarly observed in three European countries, despite considerable differences in snack food consumption, food advertisements exposure, and economic development (Vereecken et al., 2006; Currie et al., 2012; Giese et al., 2015). Furthermore, while previous studies suggested gender differences in the effects of peer norms on snack intake (De la Haye et al., 2010; Wouters et al., 2010), no systematic gender differences were found in the present study when comparing boys and girls from the same class. Different reference groups (school class vs. close friends) may account for these divergent findings, suggesting context and situation-specific modulation of social peer effects on snack intake in children and adolescents. From an environmental perspective focused on social norm effects on individual snacking behavior, the reported actual behavior of school peers is related to unhealthy snacking behavior in children and adolescents.

The present study seems to be in contrast with previous research showing no coherence between reported actual class behavior and individual unhealthy food consumption (Perkins et al., 2010). However, methodological differences may account for diverging findings. For instance, previous studies tested for additional predictive value of actual norms by controlling for perceived norms. Another difference is the closeness to the reference group (Yun and Silk, 2011). This study examined school classes as a reference group that is meaningful to the children themselves. In contrast, the actual norms determined by Perkins et al. (2010) included large reference groups (>1000), attenuating the closeness of the reference group. Future studies with systematic variations in class size may therefore reveal a gradient of effects depending on the closeness of the reference group. Furthermore, the effect of actual norms of school peers may vary for specific food items. Specifically, the present study assessed unhealthy snacking and preference for a wide range of healthy and unhealthy foods, whereas Perkins et al. (2010) focused solely on soft drinks.

Beyond demonstrating the coherence of reported actual behavior and the preferences of school peers with individual eating, the present study identified variables moderating this relationship. One finding is that adherence to class norms is moderated by individual preferences. Specifically, snack consumption increased if the class and the individual shared unhealthy preferences and decreased if they were both comparably healthy. However, and more importantly, the results showed that the relevance of class preferences for unhealthy snacking was amplified if the individual preferred unhealthy foods and was attenuated if the individual preferred healthy foods. This can be interpreted as a synergistic relationship between concurring unhealthy individual and class food preferences. The observed synergistic effect between social and individual preferences for unhealthy foods might be due to the comparably strong preference for unhealthy foods among children and adolescents whereas other reference groups like families tend to disapprove these preferences (c.f. Grube and Morgan, 1990; Birch and Fisher, 1998). Alternatively, one might assume that children and adolescents with unhealthy food preferences are somehow more prone to group norms than children and adolescents with healthy food preferences. However, the present study focused on unhealthy snacking behavior as outcome and therefore, an interesting future research question is whether also synergistic effects occur in the context of healthy snacking (e.g., fruits and vegetables intake). Overall, the results support the “contingent consistency” hypothesis (Acock and DeFleur, 1972), in that individual preferences need to be regarded in the context of normative influences, such as the school context, to explain the intake of snacks. This might be particularly important when planning interventions as one might choose to addess the class instead of the individual.

The present study also obtained evidence for the hypothesis that children and adolescents with a more positive social self-concept were more compliant with group norms. As positive social self-concept should reflect perceived acceptance by the class (Berndt and Burgy, 1996), these results also indicate that experienced inclusion facilitates class norm effects. Moreover, the results qualify a proposed positive association between unhealthy eating and popularity in schools (De la Haye et al., 2010; Stead et al., 2011). Participants with a higher social self-concept generally ate more unhealthy snacks (Table 3, step 2c), but this effect was dependent on class preferences. In addition, by differentiating individual from class preference, this study was able to take a different approach to how high self-esteem resulting from a positive social self-concept (Leary et al., 1995) is related to group adherence. The role of individual preferences for unhealthy snacking is higher for individuals with a positive compared to a negative social self-concept (Table 3, step 2d). This is unrelated to its role for class preferences.

### Limitations

The present study utilized a food frequency questionnaire (FFQ) to assess participants' unhealthy snacking behavior, and their reports were not externally verified. Although validation studies comparing food frequency questionnaires with 24 h-recall showed convergent validity down to the age of 10 (e.g., Rockett et al., 1997) the utilized FFQ only assessed the frequency but not the amount of consumption and may have underestimated the absolute amount of food intake.

Moreover, the chronological order of effects cannot be determined as the dataset is cross-sectional: For instance, rather than being the consequence of perceived group acceptance, adhering to the class norm can also contribute to acceptance by peers. Thus, children and adolescents showing high unhealthy snack consumption may be more accepted in classes with unhealthy eating norms compared to healthy ones. In similar vein, as this study does not portray dynamics, it also cannot grasp attempts to improve one's group acceptance. Hence, the results presented only complement findings of short-term group assimilation effects by experimentally induced ostracism (Williams, 1997, 2009). Generally, future adherence to group norms by people with low acceptance experience might depend on aspects such as motive for group affiliation and efficacy beliefs of group adherence in achieving inclusion (e.g., Ellemers and Jetten, 2013). This needs to be addressed in future studies.

## Conclusion

The actual norms of school peers based on behavioral reports were significantly associated with unhealthy snacking behavior in children and adolescents. Importantly, moderator variables were identified for this association. Congruent unhealthy/healthy preferences at the individual and class level increased/decreased unhealthy snack intake, respectively. This consistency effect was over-additive for unhealthy preferences. Furthermore, a positive self-concept and feelings of acceptance by classmates facilitated the effects of class norms. In sum, these results identify the role a school class might play in altering children's and adolescent's eating behavior.

### Conflict of interest statement

The authors declare that the research was conducted in the absence of any commercial or financial relationships that could be construed as a potential conflict of interest.

## Footnotes

^1^In each school, each grade is considered a separate class.
